# A DICOM Framework for Machine Learning and Processing Pipelines Against Real-time Radiology Images

**DOI:** 10.1007/s10278-021-00491-w

**Published:** 2021-08-17

**Authors:** Pradeeban Kathiravelu, Puneet Sharma, Ashish Sharma, Imon Banerjee, Hari Trivedi, Saptarshi Purkayastha, Priyanshu Sinha, Alexandre Cadrin-Chenevert, Nabile Safdar, Judy Wawira Gichoya

**Affiliations:** 1 Emory University, GA 30306 Atlanta, USA; 2 Indiana University Purdue University Indianapolis, IN Indianapolis, USA; 3 Mentor Graphics India Pvt Ltd, Noida, India; 4 Laval University, Quebec City, Canada

**Keywords:** Machine learning (ML), Picture archiving and communication system (PACS), Digital Imaging and Communications in Medicine (DICOM), Clinical data warehouse (CDW)

## Abstract

Real-time execution of machine learning (ML) pipelines on radiology images is difficult due to limited computing resources in clinical environments, whereas running them in research clusters requires efficient data transfer capabilities. We developed Niffler, an open-source Digital Imaging and Communications in Medicine (DICOM) framework that enables ML and processing pipelines in research clusters by efficiently retrieving images from the hospitals’ PACS and extracting the metadata from the images. We deployed Niffler at our institution (Emory Healthcare, the largest healthcare network in the state of Georgia) and retrieved data from 715 scanners spanning 12 sites, up to 350 GB/day continuously in real-time as a DICOM data stream over the past 2 years. We also used Niffler to retrieve images bulk on-demand based on user-provided filters to facilitate several research projects. This paper presents the architecture and three such use cases of Niffler. First, we executed an IVC filter detection and segmentation pipeline on abdominal radiographs in real-time, which was able to classify 989 test images with an accuracy of 96.0%. Second, we applied the Niffler Metadata Extractor to understand the operational efficiency of individual MRI systems based on calculated metrics. We benchmarked the accuracy of the calculated exam time windows by comparing Niffler against the Clinical Data Warehouse (CDW). Niffler accurately identified the scanners’ examination timeframes and idling times, whereas CDW falsely depicted several exam overlaps due to human errors. Third, with metadata extracted from the images by Niffler, we identified scanners with misconfigured time and reconfigured five scanners. Our evaluations highlight how Niffler enables real-time ML and processing pipelines in a research cluster.

## Introduction

There has been recent tremendous success in building machine learning (ML) models for radiology image processing tasks, including abnormality detection, segmentation, and automatic classification of pathology [1]. Radiology departments consist of several clinical systems such as picture archiving and communication system (PACS) [2] and vendor-neutral archives (VNAs) [3] that receive images in real-time from various scanners. By intercepting these images and running ML and processing pipelines on them, we can provide additional usable data to the radiologist or triage cases based on severity before they arrive on the radiologist’s worklist. However, the real-time execution of ML pipelines on radiology images is difficult as clinical systems have limited processing and memory resources to execute ML pipelines on radiology images efficiently [4, 5]. The resource scarcity hinders real-time processing of radiology images and translating advanced ML algorithms in clinical care. Therefore, we propose to shift this burden of computation away from the clinical environment and into a research platform that contains adequate computing resources and then feed these results back into the clinical environment.

The need for ML pipelines in radiology is becoming more evident in light of increased work volume and exam complexity with concurrent pressure on maintaining turnaround times for interpretations [6]. Rapid progress in the last decade in computer vision and natural language processing has ignited hope that artificial intelligence (AI) will lead to lower costs, fewer errors, more efficiency, and better health care [7]. ML pipelines have been proposed to aid in diagnosis and help with scanner optimization and effective scheduling of patients to reduce wait times [8]. Digital Imaging and Communications in Medicine (DICOM) metadata has been leveraged in identifying imaging device productivity [9]. These implementations require real-time processing of images and their metadata, which can be obtained by running computations and analytics on system performance metrics. Vendor neutral AI (VNAI) deployment infrastructure proposes fetching data from PACS and hospital information systems (HIS) to execute AI algorithms in a vendor-agnostic manner [10]. DICOM Data Warehouse (DDW) uses Mirth to parse DICOM metadata and store them in a Postgresql database [11]. Such previous works focus on enabling a research environment for ML and processing pipelines.

Several factors should be satisfied to run ML pipelines in real-time on radiology images. First, there should be a fast and secure data transfer from the PACS to research clusters where ML algorithm inference will be performed. Second, an efficient processing framework must be built to process and sort the received DICOM images and facilitate the execution of ML pipelines. Data transfer can be accomplished by leveraging the standardized DICOM format for healthcare imaging and its associated network protocol to enable a reliable transfer of imaging data and structured reports (SR) between the PACS and computing servers from data centers, clouds, and research clusters [12–14]. Although some of this work could be achieved by transferring the data and models to a cloud environment, hospital security protocols may dictate that research clusters that reside within an institutional firewall are the only permissible, secure option for healthcare images with protected health information (PHI).

This paper presents Niffler, a real-time DICOM framework that retrieves images from the PACS and extracts and processes metadata from the acquired images in the research clusters. Niffler has supported several radiology research works at our institution with its real-time and retrospective DICOM retrievals during the past 2 years. We describe three such use cases of Niffler in this paper. The first use case is an IVC (inferior vena cava) filter detection on radiology images using RetinaNet [15], on radiographs (XR, DX, CR, and DR) of the chest, spine, and abdomen (7 total exam types). We demonstrated the ability to quickly execute ML pipelines on real-time DICOM imaging data with Niffler. The second use case calculates scanner utilization by performing computations on metadata extracted from the DICOM images received in real-time. We could calculate the scanner utilization more accurately with Niffler than a clinical data warehouse (CDW) reference standard [16]. The third use case was to find the scanner clock miscalibrations by comparing the timestamps from image-receipt in the research cluster against the image acquisition time in the scanner, as indicated in the metadata. The analysis of timestamps by Niffler revealed a systematic problem in clock calibration for our scanners, which was subsequently corrected.

## Methods

We designed Niffler as a framework that retrieves DICOM images in real-time and on-demand from PACS to a research cluster. By extracting and analyzing the metadata at the research clusters, Niffler facilitates creating image subsets that can be further processed, used as data for ML and processing workflows to find valuable insights or shared with other researchers.

**Fig. 1:**
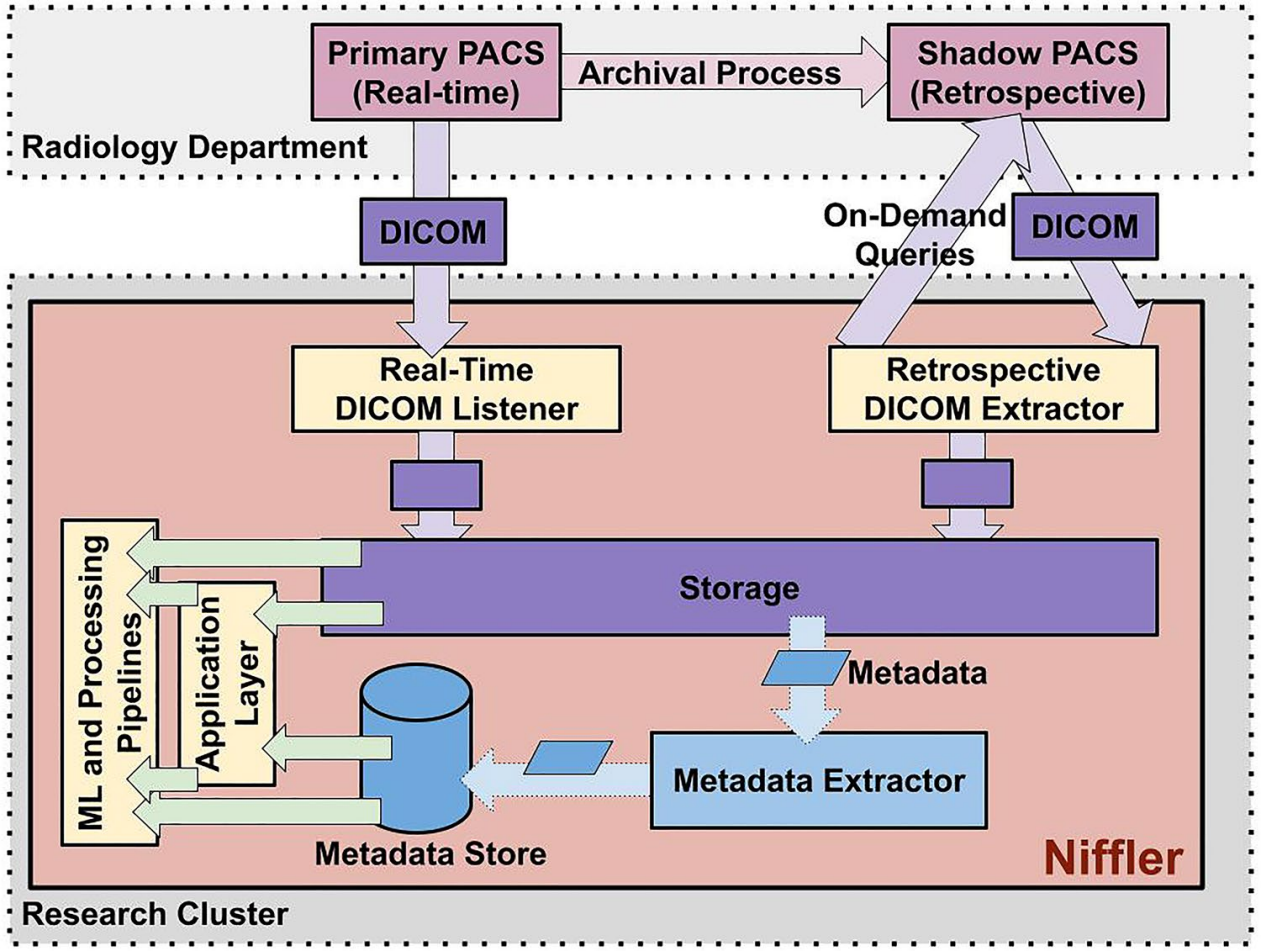
Deployment architecture. Niffler consists of DICOM listeners for receiving images in real-time, and retrospective DICOM extractors to query and retrieve images on-demand

Figure 1 depicts the Niffler architecture and prototype deployment. Niffler consists of DICOM listeners for receiving images in real-time and retrospective DICOM extractors to query and retrieve images on-demand. It also includes a *Metadata Extractor* that extracts the textual metadata from the retrieved DICOM images. Niffler stores the images in its storage and the metadata in a *Metadata Store*. Its *Application Layer* provides unified access to data and metadata in the storage and the metadata store, with several utility functions. Thus, ML and processing pipelines run efficiently on the images and metadata stored by Niffler in a research environment.

In the standard healthcare system, the radiology department may consist of several PACS, each receiving radiology images from scanners of various modalities. Our current deployment environment consists of 2 PACS from our institutional radiology department, configured to accept DICOM retrieval queries from Niffler. Niffler can receive DICOM data from more PACS systems at once, with minor configuration changes to the PACS and Niffler. In this sample deployment, the *primary PACS* receives data in real-time from the scanners. At our institution, the radiology department has configured an archival process that periodically copies the images from the primary PACS to a *shadow PACS* and then cleans up the primary PACS every week. Hence, the shadow PACS stores imaging data for several years, supporting retrospective queries. We deployed Niffler in a standard server with 12 GB memory, AMD Opteron 63xx class CPU, and 1 TB of local hard disk in the research cluster. As we started to extract more data on-demand, we later attached a 32 TB network drive as additional storage. We also expanded to leverage our research cluster for a distributed execution of the pipelines.

Niffler enables the execution of ML pipelines as Docker [17] containers on DICOM images retrieved from both PACS and the textual metadata of the images. The pipelines are run either through WDL specifications with a workflow manager that we developed extending Cromwell [18] or by simple shell scripts. The *Real-Time DICOM Listener* receives images from the primary PACS continuously as a DICOM imaging stream. The *Retrospective DICOM Extractor* parses a user-provided CSV file consisting of data that needs to be extracted from the PACS, as specified in a JSON [19] configuration file. The CSV file may have a list of EMPI, {EMPI, Accession}, Accession, or {EMPI, StudyDate}. Niffler parses the CSV file to make a series of C-FIND and C-MOVE queries on the shadow PACS. Niffler avoids the need to manually execute several C-MOVE or C-FIND and C-MOVE queries to extract the images. Niffler consists of multiple DICOM StoreSCP processes configured at the research cluster, one process for each PACS. It stores the images from the PACS separately in encrypted storage, in a hierarchical folder structure. By default, this structure is patient-folder/study-folder/series-folder/instance.dcm. However, this storage structure (together with several other Niffler system and configuration properties) can be configured through JSON configuration files. For example, images can also be stored in various levels of hierarchy (such as patient-folder/instance.dcm or patient-folder/study-folder/instance.dcm).

The *Metadata Extractor* traverses and queries all the images in the storage, extracts the relevant metadata from the DICOM headers, and stores the PHI-free metadata in a NoSQL [20] database, which we call the *Metadata Store*. The NoSQL database is chosen due to its scalability and support for data storage as JSON [19, 21] documents, which natively suits the hierarchical format of DICOM metadata. The database consists of several *collections* (i.e., database tables), each representing a *profile*. Each profile defines the DICOM attributes that must be extracted for one or more experiments. The Metadata Extractor reads the folder consisting of the *profiles* stored as text files. It parses the DICOM headers from the images received in the storage and stores the relevant attributes defined in the profiles into their respective collections. An experiment can use existing profiles or create a new profile at run time without halting the execution. As each profile generates a collection, the access to the metadata store can be limited to the respective researcher at the collection level, using the access controls offered by the database. The metadata is used to filter cohorts and subcohorts that allow dataset creation for model inference. For example, to test whether an IVC filter model performance drops with the change of equipment, cohorts of data filtered by modality and manufacturer are easily created at the metadata level.

The *Application Layer* facilitates access to the DICOM images from the storage and the respective metadata from the metadata store. It provides unified data access to both data and metadata. It also offers utility functions such as de-identification and image conversion and scripts such as scanner utilization computation and scanner clock calibration. The ML pipelines run their algorithms, either directly or via the application layer, on the images and the metadata. The services such as de-identification and image conversion are optimized to run using multiple processes as well as on a cluster by using Slurm.

Niffler deletes the images from the storage periodically once the metadata extraction and the execution of the ML pipelines on the images are complete. Subsets of images relevant for a study can be shared with the other researchers, typically after processing them, including de-identifying images, converting DICOM images into PNG, or the image output with associated ML inference results. Niffler facilitates the users to determine the cohort components required for model inference. Since the pipeline is a prospectively populated system with an option for a query to extract images meeting a specific criterion, this limits the duplicated information stored in the research clusters.

Currently, with existing open-source and enterprise frameworks, a researcher would have to submit multiple queries to the PACS and CDW manually, work on anonymizing the data collected, merge the data, and then run the model inference. Niffler automates this entire process and thus minimizes the human-in-the-loop. Niffler supports prospective dynamic cohort and subcohort creation, eliminating the need for duplicate data storage and aggregation with anonymized model output. Through its native support for the ML and processing pipeline execution as containers, Niffler provides an infrastructure-agnostic execution with seamless scaling and migration. Thus, Niffler minimizes the repetitive and complicated configuration steps while automating the end-to-end process of an ML pipeline.

### Niffler Execution

Niffler is configured as a system process with its storage and metadata extraction functionality. At the core of the Metadata Extractor is an *extract_metadata* process that runs continuously in a loop. The extract_metadata runs every 10 min by default but can be configured to run as soon as the Niffler real-time DICOM listener receives an image. Niffler stores the DICOM images in the file system by default. Hence, the Metadata Extractor uses the *find* operating system command as a sub-process to traverse all the DICOM series in the storage. In each iteration, the Metadata Extractor extracts metadata from the first image of each series that is not extracted yet. For performance reasons, Niffler extracts metadata from only one image per series. However, we can configure it to extract more than one (such as first, last, and a middle instance in any given series) or all the images of each series.

Niffler has a *clear_storage* process that runs nightly (by default, at 23:59). It deletes the images whose metadata are already extracted, making sure no ML or processing workflow is still processing them. Niffler periodically stores the progress of *extract_metadata* and *clear_storage* processes to the filesystem as sets of series identifiers. By writing the sets to the filesystem and reading them upon startup ensures that the Metadata Extractor processes resume where they stopped. As Niffler is written in Python, the progress of operations such as metadata extraction, on-demand extraction, and image conversion are stored in pickle files, allowing halt-and-resume of the progress from a long list of DICOM instances. This approach aims to support seamless updates and improve fault tolerance, ensuring that the progress made by the extraction and deletion processes is not lost upon failures and restarts. Hence, when Niffler is restarted manually or due to external measures (such as a system restart), it resumes where it left off during the previous iteration.

### Implementation

We developed the Niffler prototype as an open-source platform,^1^ with its core and utility functions developed in Python3. The prototype uses the Pydicom library to extract metadata and process the DICOM images (including converting the DICOM images to PNG format and converting DICOM images into anonymized DICOM images with only a subset of metadata attributes stored as headers) and MongoDB [22] as its NoSQL Metadata Store. The application layer consists of several toolkits. Among these, the scanner utilization is in Java. Scanner clock calibration is in Javascript. The image converter and anonymizer are in Python.

Niffler supports the ML and processing pipelines in different coding languages, provided that the pipeline can be wrapped as a container. Two instances of the DCM4CHE [23] StoreSCP tool are configured to receive all images in 2 different ports. The first one listens to all the images sent in real-time from the primary PACS and accepts them. The second one retrieves specific images retrospectively on-demand from the shadow PACS via a series of DCM4CHE MoveSCU queries, which are optionally first filtered based on FindSCU queries — all internally through Niffler, as such the user can retrieve thousands of images belonging to various patients and accessions (stored in a CSV file) with a single Python command of Niffler. We deployed Niffler in a server secured by strict firewall rules and configured the MongoDB metadata store with authentication. For data transfer efficiency, Niffler supports receiving data in a secure compressed DICOM data stream. In our sample deployment, the images received from the PACS are in JPEG lossless compressed form. Niffler uses GDCM [24] to export the compressed DICOM images to a PNG format for the ML pipelines to consume the images in a de-identified manner.

## Results

We demonstrated the capability and stability of Niffler by running it continuously over 2 years, receiving images from the two PACS, including up to 350 GB/day of real-time data from 715 scanners spanning 12 institutions. Our research lab, composed of 57 members (radiologists, faculty, postdocs, and research students), extensively used Niffler to query and retrieve retrospective data on-demand for more than a year, in addition to this ongoing real-time data retrieval. The on-demand DICOM retrieval time depends on the total volume (influenced by the network latency between the research environment and the radiology network) and the number of files (DICOM retrieval queries for each accession, thus leading to more time for each C-FIND and C-MOVE requests). The volume of a DICOM image and how many image instances in a series both heavily depend on the modality. The performance also heavily depends on the load of the PACS. Our on-demand extractions are not mission-critical and are of lower priority than the clinical extractions from the PACS. Therefore, the PACS is configured to lower our on-demand extraction speed when there is a higher load/demand on the PACS. We retrieved images of various modalities retrospectively, including CT, MR, DX, and several others.

We retrieved more than 50 TB in a month of mammography images, consisting of around 3.3 million images. Each cohort of DICOM retrieval was around 1.4 TB. Niffler retrieved approximately 1.5 TB of mammography per day consistently, often extracting a bunch of 4.5 TB in 3 days. Furthermore, 4 months (Jan–April 2020) of CXR data from PACS took 30 h to retrieve. This extraction consisted of 58,298 accessions with 1 TB volume (226,348 directories, 158,445 files). On another occasion, we retrieved CXR images at a pace of 160,000 images in 5.4 h.

We measured the performance of Niffler with three practical use cases. First, we built an IVC filter (IVCF) detection pipeline as a container to execute on the images retrieved in real-time with Niffler. The pipeline uses the Keras RetinaNet object detection pre-trained model to determine whether an IVCF is detected in the subcategories of the images. The backbone encoder CNN was based on the Resnet-50 architecture [25] pre-trained on the COCO object detection dataset [26]. The model was trained on 503 abdominal, thoracoabdominal, and lumbar radiographs from various projection views and validated on 127 images.

**Fig. 2:**
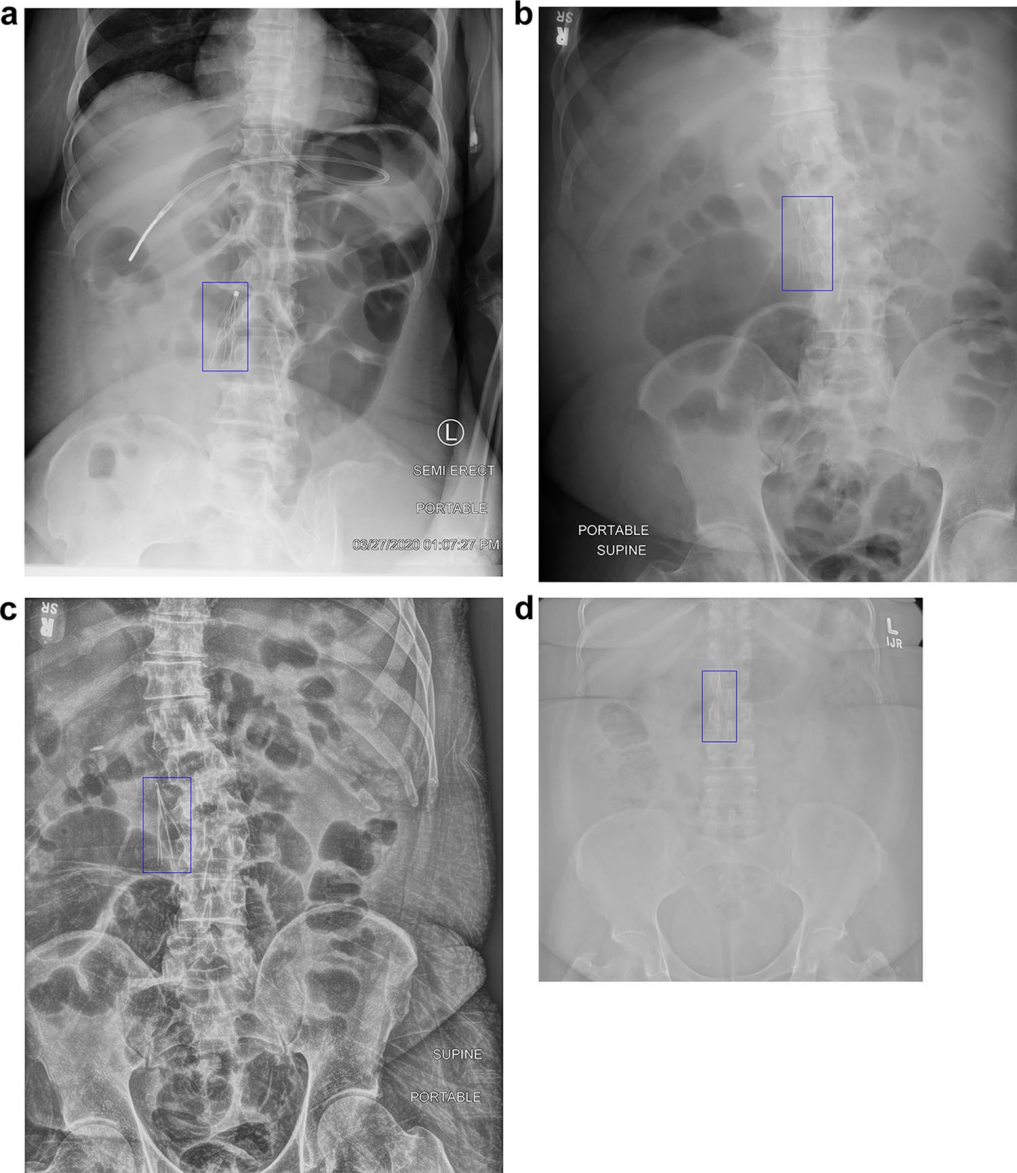
IVCF detection and localization on various views. The pipeline draws a bounding box around the identified IVCF in the images and outputs a PNG image with the detection box

Niffler yielded real-time detection of IVC filters on these radiographs with an average end-to-end latency (measured as the time difference between when a scanner acquires an image and when the ML pipeline processes it) of 20 min and high model accuracy. During the real-time inference, the Niffler Metadata Extractor applied the filters on modality and body parts to create a DICOM subset consisting of 989 DICOM images. The IVCF detection container ran its inference on the identified images, including chest X-ray, abdomen radiographs, and Spine X-rays. The pipeline drew a bounding box around the identified IVCF in the images and output a PNG image with the detection box, as shown in Fig. 2. Two interventional radiologists reviewed all the outputs and determined that the IVCF detection algorithm classified the test images with high accuracy of 96.0% on the test images.

**Fig. 3:**
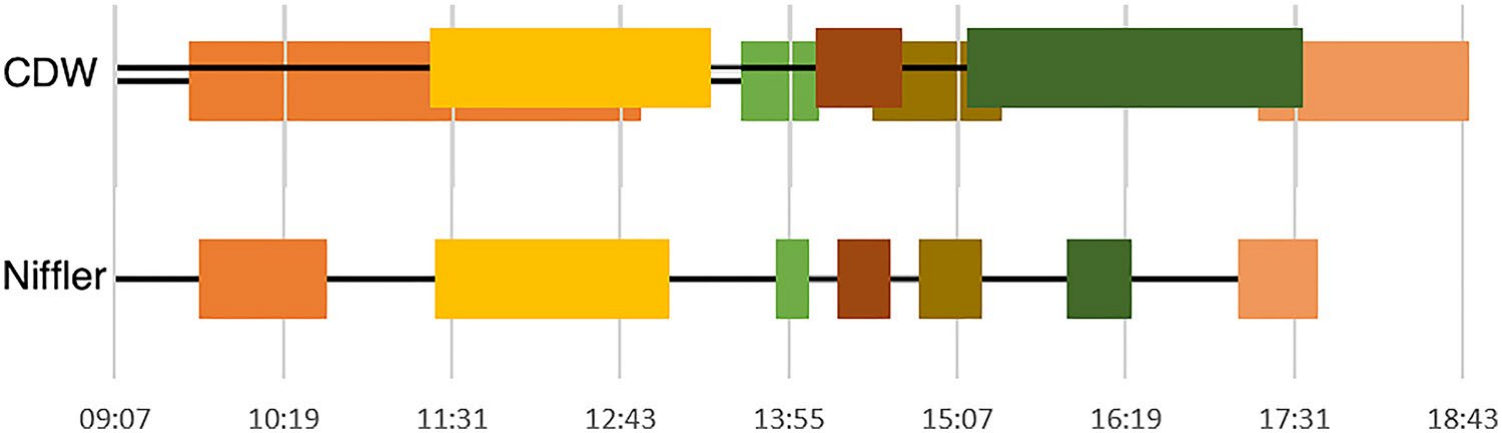
Visualizing scanner utilization measurements. We observe calculated exam time windows from one scanner on a particular day, with Niffler and CDW

For the second use case, we applied the Niffler Metadata Extractor to understand the operational efficiency of individual MRI systems based on calculated metrics from exam timestamps. These data allow measurement of exam duration and system idle time. Figure 3 indicates the calculated exam time windows from one scanner on a particular day, according to Niffler and CDW.

Identifying scanner utilization from CDW is more prone to human errors, leading to a false depiction of exam overlap. The overlapping timelines for the exams indicated by CDW are not even possible as a scanner can perform only one exam at any given time frame. Niffler is free from such human errors as it identifies the image acquisition times directly from the tags in the metadata of the images’ DICOM headers. As such, Niffler consistently and accurately identified exam timeframes and idling times of the scanners. Our evaluations on Niffler highlight the feasibility and efficiency in running the ML and processing pipelines from a research cluster on the images and metadata received in real-time and retrospectively from hospital PACS.

Finally, as a third use case, we used Niffler to identify the scanners with misconfigured time, using the metadata of the images received in real-time. When metadata attributes such as AcquisitionTime and SeriesTime have a wrong timestamp due to the scanner time misconfiguration (typically, due to a wrong timezone setting), extracting useful information from the metadata becomes harder. Niffler contains a script that compares the time the images are received at the metadata store against the acquisition time from the metadata. The difference should not exceed 20 min, considering the time the image to be received over the network and the metadata to be extracted and saved. Niffler correctly identified five scanners with misconfigured time, which were subsequently reconfigured. IHE Consistent Time [27] profile ensures computers in a healthcare network have their times synchronized. However, in practice, a vast healthcare network spanning multiple sites (such as our healthcare network that spans 12 sites and 715 scanners) can have misconfigured time and time zones, as we observed. Hence, we note that Niffler can detect such anomalies by processing the metadata.

## Discussion

We develop and maintain *Niffler*, an open-source DICOM framework that retrieves images from the radiology PACS using DICOM network listeners and supports the execution of ML pipelines formatted as containers on radiology data. Niffler uses the DICOM standard to receive images in real-time and on-demand based on user-specified queries from the PACS. In the real-time case, the images are sent from scanners to the primary PACS and then from the primary PACS to Niffler real-time DICOM listener as they arrive. This process incurs end-to-end latency of up to 20 min due to the network delays and processing by the Niffler metadata extractor. As Niffler receives an image sent by a PACS in real-time and extracts the metadata in 20 min, it enables pseudo real-time ML and processing pipelines in practice. Furthermore, Niffler can be configured to perform the metadata extraction more frequently (instead of 10 min intervals), thus shortening this time interval further down. All PACS environments support DICOM networking. As such, Niffler is compatible across all PACS environments with no additional vendor-specific configurations.

Our evaluations highlight that Niffler provides fast processing of ML models in real-time. Niffler achieves high efficiency by running the filtering at the metadata level and the ML pipelines using CPU only on the identified images. We also highlight the potential in using Niffler to operate the scanners better, with information not otherwise readily available in the clinical systems such as PACS and CDW. The prototype and the evaluations highlight the potential and performance of Niffler in executing ML and processing pipelines in real-time and retrospectively. With minimal tuning of infrastructure, Niffler will facilitate the execution of ML models from any standard radiology environment. Niffler further enables the development of models against real-time data streams and helps gather large-scale prospective data in a centralized store to facilitate imaging research.

Niffler enables a research sandbox, connecting clinical data flows in real-time and historical data from the PACS in a research cluster. As a pilot center for the ACR AI lab, we integrate this pipeline for AI annotation, training, and inference. We aim to deploy clinically validated algorithms on DICOM data and metadata retrieved from the PACS using Niffler while also enabling sending the results from running the algorithms back into the clinical systems. In future work, we aim to extend Niffler to facilitate feedback into the clinical systems from the algorithms validated in the Niffler research cluster environment.

Our Niffler deployment’s real-time DICOM listener continues to receive around 350 GB/day of data for more than 2 years, all the images that the primary PACS receives without a filter. Niffler also efficiently extracted the metadata from the acquired images. Besides, it also efficiently handled retrospective queries from the shadow PACS on the historical data. However, we note that if we configure Niffler to receive real-time images from several PACS (rather than just 2) on the same scale, a stand-alone deployment of Niffler will face bottlenecks in data transfer or metadata extraction. This prediction prompted us to invest time in investigating such executions based on a cluster. Niffler can be deployed in a distributed manner in a cluster to support the processing of data acquired from several PACS. In addition to the deployment presented in this paper, we have also currently deployed Niffler in three other environments: another lightweight VM similar to the one presented in this paper and two larger servers. They are all running various ML and processing pipelines of our research team. We believe the proposed approach and the Niffler open-source framework will help the radiology research community at large.

## Conclusion

In this paper, we presented Niffler, an open-source DICOM framework that supports the seamless transfer of data from the PACS to the research clusters and enables efficient execution of ML and processing pipelines on the images, reports, and the extracted textual metadata. Niffler facilitates the execution of ML models with a minimal tuning of infrastructure. It further enables the development of models against real-time data streams and helps gather large-scale prospective data in a centralized store to facilitate imaging research. We demonstrated the potential for seamless execution of ML and processing pipelines in real-time with three use cases of Niffler — one on ML workflows on the DICOM images and the other two by processing the extracted metadata.

Niffler requires extension and further development for clinical validation. In the IVCF detection, we currently do not know if the patient is anticoagulated and can have this filter removed, they have contraindication of filter removal, or already have an upcoming scheduled appointment for filter retrieval. As future work, we propose to support end-to-end clinical validation of the ML pipelines with the consumption of electronic medical record (EMR) from the real-time analytics (RTA) on the laboratory information (INR, anticoagulation profile), medications (whether the patient is on any anticoagulant), problem list (for example, if a patient has a history of GI bleed and hence cannot be anticoagulated), and the upcoming clinical appointments where a patient can be seen in the clinic. Linkage to an HL7 [28] ADT (admission, discharge, and transfer**) **message would allow just-in-time clinical review of the patients in same-day appointments. In the IVCF detection pipeline, such a linkage will provide education to providers on the benefits of the IVCF removal when no longer required.

## Footnotes

1 https://github.com/Emory-HITI/Niffler.

## Declarations

### Statement of Data Access and Integrity

The authors declare that they had full access to all of the data in this study and the authors take complete responsibility for the integrity of the data and the accuracy of the data analysis.
